# Exposure to neonicotinoids influences the motor function of adult worker honeybees

**DOI:** 10.1007/s10646-014-1283-x

**Published:** 2014-07-11

**Authors:** Sally M. Williamson, Sarah J. Willis, Geraldine A. Wright

**Affiliations:** 1 Faculty of Medical Sciences, Institute of Neuroscience, Newcastle University, Newcastle upon Tyne, NE1 7RU UK; 2 School of Biology, Newcastle University, Newcastle upon Tyne, NE1 7RU UK

**Keywords:** Honeybee, *Apis mellifera*, Neonicotinoid, Pesticide, Pollinator, Imidacloprid

## Abstract

Systemic pesticides such as neonicotinoids are commonly used on flowering crops visited by pollinators, and their use has been implicated in the decline of insect pollinator populations in Europe and North America. Several studies show that neonicotinoids affect navigation and learning in bees but few studies have examined whether these substances influence their basic motor function. Here, we investigated how prolonged exposure to sublethal doses of four neonicotinoid pesticides (imidacloprid, thiamethoxam, clothianidin, dinotefuran) and the plant toxin, nicotine, affect basic motor function and postural control in foraging-age worker honeybees. We used doses of 10 nM for each neonicotinoid: field-relevant doses that we determined to be sublethal and willingly consumed by bees. The neonicotinoids were placed in food solutions given to bees for 24 h. After the exposure period, bees were more likely to lose postural control during the motor function assay and fail to right themselves if exposed to imidacloprid, thiamethoxam, clothianidin. Bees exposed to thiamethoxam and nicotine also spent more time grooming. Other behaviours (walking, sitting and flying) were not significantly affected. Expression of changes in motor function after exposure to imidacloprid was dose-dependent and affected all measured behaviours. Our data illustrate that 24 h exposure to sublethal doses of neonicotinoid pesticides has a subtle influence on bee behaviour that is likely to affect normal function in a field setting.

## Introduction

Many of the world’s crops are pollinated by insects, with 35 % of global food production depending on animal pollination services (Klein et al. 2007). However, many countries have experienced a loss of insect pollinators in recent years, a situation which threatens ecological stability and global food security (Calderone 2012; Garibaldi et al. 2009). Honeybee numbers are in decline, with countries in both Europe and North America reporting recent heavy losses of honeybee colonies (Mutinelli et al. 2010; Vanengelsdorp et al. 2008). It is likely that these losses result from a combination of factors, including loss of wildflowers, disease and parasites, and even exposure to chemical treatments which are used to combat parasites (Desneux et al. 2007; Dainat et al. 2011; Hawthorne and Dively 2011). Honeybees are exposed to many different agricultural chemicals as they forage on the flowers of treated crops (Mullin et al. 2010). Although it is true that potential exposure limits for most chemicals are not directly lethal to bees, sublethal doses of certain chemicals can adversely affect bees in ways which can affect colony fitness (Mullin et al. 2010; Wu et al. 2011).

Neonicotinoid pesticides in particular have been implicated in honeybee decline (Maxim and van der Sluijs 2010). These pesticides are synthetic compounds, structurally similar to nicotine, which target insect nicotinic acetylcholine receptors (Millar and Denholm 2007). There are several classes of neonicotinoid that are based on their chemical structures: chloronicotinyl compounds, such as imidacloprid; thianicotinyl compounds, such as clothianidin and thiamethoxam; and the furanicotinyl compound dinotefuran (Millar and Denholm 2007). More recently, cyano-nicotinyl compounds such as acetomiprid and thiacloprid have also been introduced to agriculture. Both nicotine and imidacloprid act as partial agonists of insect neuronal nAChRs (Deglise et al. 2002), whereas clothianidin is a more potent compound which acts as a superagonist (Brown et al. 2006).

Neonicotinoids are known to affect many aspects of honeybee behaviour. Imidacloprid has been most widely studied, and its adverse effects on olfactory learning and memory have been well established (Decourtye et al. 2004a, b; Williamson and Wright 2013) as well as visual learning (Han et al. 2010). Imidacloprid affects gustatory sensitivity to sucrose, and also impairs the ability of honeybees to perform the waggle dance (Eiri and Nieh 2012; Lambin et al. 2001), perhaps suggesting that they also impair motor function. One study, on the other hand, reported that sublethal doses of imidacloprid do not impair motor function of the proboscis extension response (Ramirez-Romero et al. 2008). Imidacloprid, clothianidin and thiamethoxam have all reduced the ability of bees to forage and perform homing flights in field situations (Bortolotti et al. 2003; Mommaerts et al. 2010; Schneider et al. 2012; Fischer et al. 2014). At high acute doses (100 and 500 ppb) imidacloprid affects locomotion (Medrzycki et al. 2003), although such concentrations far exceed relevant field concentrations (Blacquiere et al. 2012). Few studies have investigated the more subtle effects which neonicotinoids may have on motor function behaviour, when administered at field realistic doses over a 24 h period, simulating a bee’s exposure in the field to a large acute dose; nor has any previous study directly compared the effects of all classes of neonicotinoid on honeybee behaviour (Decourtye et al. 2013).

Here we report the effects of oral exposure to nicotine and four different neonicotinoid compounds on honeybee motor function behaviours. The behaviours include walking, flying, grooming, remaining still and falling upside down, and were measured in an assay using the Noldus Observer software, using methods modified from Maze et al. (2006).

## Materials and methods

### Honey bee capture and exposure to pesticides

Mixed age, foraging, adult worker honeybees (*Apis mellifera* var. Buckfast) from a population bred at the National Bee Unit (FERA, Sand Hutton, UK) were collected from an outdoor colony maintained at Newcastle University from June to September 2012. All of the bees used in the experiment were from the same colony. The colony had evidence of varroa infestation and was treated once with oxalic acid to control varroa after the experiments were conducted. Honeybees were collected in small cylindrical plastic containers, cold anaesthetised on ice, and transferred into 16.5 × 11 × 6.5 cm^3^ plastic boxes. Three 2 ml microcentrifuge tubes with four evenly spaced 2 mm holes were filled with 1.0 M sucrose solution containing a pesticide and pushed through holes in the sides of the boxes. Fifteen adult worker bees were placed into each box and left at room temperature to feed ad libitum on the pesticide solutions for ~24 h.

### Pesticides

We used four neonicotinoid pesticides (dinotefuran, thiamethoxam, imidacloprid, clothianidin) and nicotine obtained in powdered form from Sigma-Aldrich. Stock solutions of 1 mM were made in 1 M sucrose solution and diluted in series with 1 M sucrose to the appropriate concentration. Experimental solutions were re-made daily from frozen stock solutions. A pilot study with the concentrations of 10 and 100 nM was run to identify that all pesticides were being used at a sublethal dose (as defined by Desneux et al. 2007). In this pilot study, we used 4 cohorts of 15 bees per treatment. The total amount of sucrose solution consumed and the number of bees alive was measured 24 h later. The amount of solution was obtained by weighing the food tubes before and after 24 h. The volume consumed per bee was derived by dividing the weighed value per tube by the density of a 1 M sucrose solution (1.13). To compare our study to other reports of the influence of neonicotinoids on bees, we constructed a table with the estimated average dose of each solution (Table 1). (*Note* The ng/ml calculation in Table 1 was not adjusted for the density of each neonicotinoid because the contribution of the neonicotinoid to the weight of each solution was negligible at nM concentrations.)

**Table 1:** Comparison of doses consumed per bee for data in Fig. 1b

	10 nM				100 nM			
	PPB	ng/g or ng/ml	Mean vol consumed (ml)/bee/24 h	ng/bee/24 h	PPB	ng/g or ng/ml	Mean vol consumed (ml)/bee/24 h	ng/bee/24 h
Nicotine	1.62	1.62	0.155	0.252	16.2	16.2	0.156	2.54
Imidacloprid	2.56	2.56	0.156	0.401	25.6	25.6	0.144	3.70
Thiamethoxam	2.92	2.92	0.164	0.481	29.2	29.2	0.124	3.62
Clothianidin	2.50	2.50	0.137	0.344	25.0	25.0	0.119	2.99
Dinotefuran	2.02	2.02	0.160	0.323	20.2	20.2	0.143	2.89

*Note* ng/bee/24 h values were calculated as the product of ng/ml and the mean volume consumed/bee/24 h

A 10 nM dose of all pesticides was selected for use in the motor function assays, as this did not increase mortality in any of the compounds tested and was in the range of the reported values from field collected nectar and pollen (Blacquiere et al. 2012; Sanchez-Bayo and Goka 2014). Six cohorts of 15 bees were collected as above and fed with a treatment solution for 24 h; each box contained a different treatment. Each treatment including a control was run simultaneously to ensure that variability due to season or cohort was spaced across treatments. A subset of bees (*N* = 3) were used from each box for the behavioural assay. In a separate experiment to test whether there was an influence of dose on motor function, we used three concentrations of imidacloprid (10, 100 nM and 1 µM). The same procedure was used as above.

### Behavioural assay

After 24 h of pesticide administration, individual bees were removed from the box and placed into 150 × 15 mm^2^ petri dishes and observed in an assay originally described in Maze et al. (2006). The petri dishes each contained a piece of moistened paper towel to maintain humidity and had holes drilled into the top for ventilation. The bees were extracted from the boxes by placing a cylindrical plastic container over a hole in either the top or the side of the box and allowing the bees to climb into these. If the bees were unable to climb into these containers, they were extracted using forceps. The bee was left to acclimatise for 1 min, its behaviour was observed continuously for 15 min. Behavioural observations were recorded using the Noldus Observer software (Noldus Information Technology, Wageningen, Netherlands). The five behaviours quantified were walking, flying, remaining still, falling upside down, and grooming (Table 2); these behaviours have previously been shown in our laboratory to be affected by pesticide exposure or drug administration (Maze et al. 2006; Williamson et al. 2013). Each behaviour was coded as a discrete state and the observation recorded behaviour continuously over the 15 min interval by the person doing the observation (SJW).

**Table 2:** Behaviours measured in the locomotion assay

Behaviour	Description relative to a bee
Walking	Walking around the petri dish including along the sides or the top
Flying	Flying around the petri dish or into the side or top of it
Still	Not moving but upright
Upside down	Lying on its back and either remaining still or moving its wings, failing to turn over or remain upright
Grooming	Grooming of any body part

### Statistical analysis

Data analysis was conducted using IBM SPSS (v.19). Generalized linear models (GLZM) were used to model the percentage of the interval and mean duration data using the Tweedie distribution with an identity link function for the tests involving all neonicotinoids; for the tests against imidacloprid alone, a Tweedie model with a log link function was used. Tweedie models were selected due to the bimodal distribution of the data. Poisson regression (Preg) models were fit to the bout data and the mortality data. A generalized linear model with a scale response variable was used to analyse the food consumption data. Least-squares contrasts (lsc) were used to make multiple comparisons throughout; multiple comparisons were not performed unless there was a significant main effect.

## Results

### Identification of sublethal doses of neonicotinoids

**Fig. 1:**
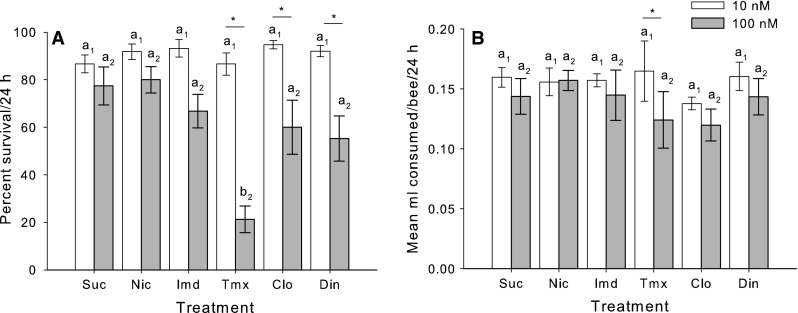
Identification of sublethal concentrations and consumption of solutions. **a** Mortality was unaffected by ad libitum consumption of 10 nM neonicotinoid solutions; of the 100 nM solutions, only thiamethoxam increased mortality. **b** Bees consumed slightly less of the 100 nM concentration of the neonicotinoids in 1 M sucrose solution on average. Post hoc comparisons against the control (sucrose) are indicated by letters (e.g. ‘*a*’); the number indicates the treatment (*a*_1_ = 10 nM, *a*_2_ = 100 nM). The* asterisk* indicates a significant (*P* < 0.05) pairwise, post hoc comparison of 10 versus. 100 nM for each neonicotinoid treatment. *Note* a separate sucrose control group was performed for each concentration neonicotinoid. *Suc* sucrose (control), *Nic* nicotine, *Imd* imidacloprid, *Tmx* thiamethoxam, *Clo* clothianidin, *Din* dinotefuran. *N* = 4 cohorts of 15 bees for each treatment group. *Bars* represent means ± SE

To identify a sublethal dose, we did a pilot experiment which compared the mortality of the bees over a 24 h period when fed with either a 10 or a 100 nM dose of each pesticide in sucrose solution. Bees fed the 100 nM dose were on average more likely to die overnight than those fed the 10 nM dose but this depended on the pesticide (Fig. 1a, Preg, concentration x treatment, χ_5_^2^ = 15.0, *P* = 0.010). At 24 h, only bees fed 100 nM thiamethoxam had significantly higher mortality than the sucrose control (lsc, *P* < 0.001). Bees fed thiamethoxam, clothianidin and dinotefuran had significantly greater mortality when the doses were compared for each pesticide (all pairwise lsc, *P* < 0.05). The total consumption of the solution over 24 h was slightly lower on average for the bees fed the 100 nM dose of all pesticides (Fig. 1b, GLZM, concentration main effect, χ_5_^2^ = 5.09, *P* = 0.024), but it did not depend on which pesticide treatment the bees were fed (GLZM, treatment main effect, χ_5_^2^ = 5.48, *P* = 0.360).

### Comparison of the effects of neonicotinoids on behaviour

**Fig. 2:**
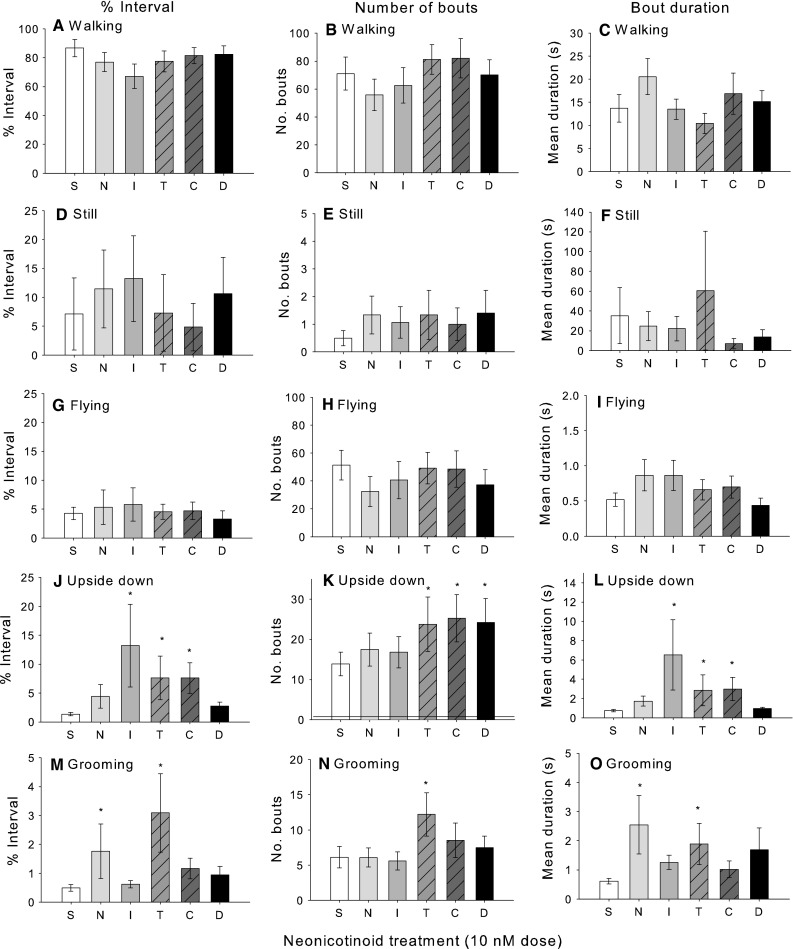
Effects of 10 nM doses of neonicotinoids on behaviour. Walking behaviour (**a–c)**, time sitting still (**d** ***–*** **f)**, and flying behaviour (**g** ***–*** **i)** were not significantly different among pesticide treatments. Exposure to imidacloprid, thiamethoxam, and clothianidin influenced the time spent upside down (**j)**. The number of bouts of upside down behaviour (**k)** was affected by thiamethoxam, clothianidin, and dinotefuran, whereas imidacloprid, thiamethoxam, and clothianidin influenced mean bout duration of upside down behaviour (**l)**. The time spent grooming was affected by nicotine and thiamethoxam (**m)**. The number of grooming bouts was greater in thimethoxam treated bees (**n)** and mean grooming bout duration was longest in nicotine and thiamethoxam treated bees (**o)**. *S* sucrose, *N* nicotine, *I* imidacloprid, *T* thiamethoxam, *C* clothianidin, *D* dinotefuran. *N* = 15 individual bees for each treatment group. The* asterisk* indicates a significant (*P* < 0.05) pairwise, post hoc comparison for each neonicotinoid treatment to the control (sucrose). *Bars* represent means ± SE

When placed in the assay within the Petri dish, bees spent on average ~80 % of their time walking (Fig. 2a–c), 5–10 % of the time standing still (Fig. 2d–f) and less than 5 % of the time trying to fly (Fig. 2g–i). The 10 nM doses of the neonicotinoids or nicotine did not significantly alter the walking, flying, or standing still behaviour (% of time spent, number of bouts, or mean bout duration) of adult forager honeybees (Fig. 2a–i; Table 3). However, if the bees had been fed solutions containing thiamethoxam, imidacloprid, clothianidin or nicotine, they were more likely to lose postural control and spend more time laying on their backs, unable to right themselves (Fig. 2j–l; Table 3). The mean number of bouts of behaviour was also greater for bees exposed to thiamethoxam, clothianidin, and dinotefuran (Fig. 2k) and the mean duration of each bout longer for bees exposed to imidacloprid, thiamethoxam, and clothianidin (Fig. 2l). Thiamethoxam and nicotine also caused bees to spend more time grooming (Fig. 2m; Table 3). Thiamethoxam caused more bouts of grooming (Fig. 2n), and thiamethoxam and nicotine caused longer mean duration of grooming bouts (Fig. 2o).

**Table 3:** GLZM test statistics for the effects of neonicotinoids on behaviour

	% interval		Bouts		Mean duration	
	χ_5_^2^	*P* value	χ_5_^2^	*P* value	χ_5_^2^	*P* value
Walking	3.00	0.700	3.5	0.624	7.81	0.167
Still	1.35	0.929	0.35	0.986	4.40	0.493
Flying	2.25	0.814	2.16	0.827	5.42	0.367
Upside down	35.4	<0.001	6.46	0.264	31.7	<0.001
Grooming	23.1	<0.001	6.59	0.254	19.4	0.002

### Comparison of imidacloprid concentrations on behaviour

**Fig. 3:**
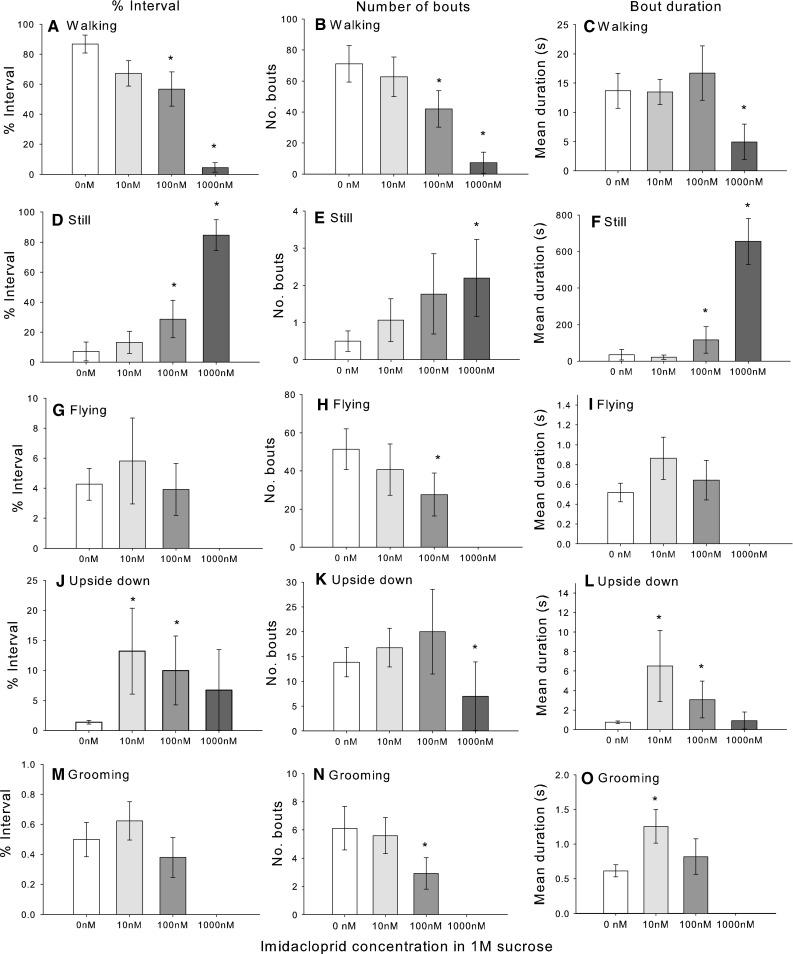
The effect of imidacloprid exposure on motor function is concentration-dependent. High doses of imidacloprid reduced walking behaviour (**a** ***–*** **c)**, increased the time spent standing still (**d** ***–*** **f)**, and completely abolished flight behaviour (**g** ***–*** **i)**. Bees given intermediate concentrations (10 and 100 nM) of imidacloprid spent more time upside down (**j)** and had longer bouts of upside down behaviour (**l)**. High doses of imidacloprid completely abolished grooming behaviour (**m)** and reduced the number of bouts of grooming (**n)**. A 10 nM concentration increased the mean grooming bout duration (**o)**. *N*_S_ = 16, *N*_10nM_ = 15, *N*_100nM_ = 13, *N*_1µM_ = 10. *X-axis* is the concentration of imidacloprid in 1 M sucrose solution. The* asterisk* indicates a significant (*P* < 0.05) pairwise, post hoc comparison for each neonicotinoid treatment to the control (sucrose). *Bars* represent means ± SE

To identify whether the concentration of the neonicotinoids influenced locomotion after 24 h exposure, we tested three different concentrations (10, 100 nM, 1 µM) of imidacloprid in the same assay described above. In general, bees exposed to imidacloprid for 24 h exhibited a dose-dependent reduction in walking (Fig. 3a–c; Table 4) and an increase in the time spent still (Fig. 3d–f). Exposure to 10 or 100 nM doses of imidacloprid did not affect time spent flying (Fig. 3g–i) but the bees fed the 1 µM dose never exhibited flying (to fit the model, it was necessary to exclude these bees, Table 4). Imidacloprid exposure also affected the time spent upside down (Fig. 3j–l). Bees fed 10 and 100 nM imidacloprid spent significantly more time upside down, indicating that they had difficulty performing the righting reflex, whereas bees fed the 1 μM were not significantly different to the control. Imidacloprid also affected grooming in a concentration dependent manner, with high doses inhibiting grooming (Fig. 3m–o). Furthermore, with the exception of two outliers that had mean bout durations between 20 and 30 s long, none of the bees treated with 1 μM imidacloprid exhibited grooming (Fig. 3m–o; Table 4). (The outliers were taken out of the analysis reported here—to fit the models, we also excluded the 1 μM bees from the analysis as for flying behaviour.)

**Table 4:** GLZM test statistics for the effects of imidacloprid concentration on behaviour

	% Interval		Bouts		Mean duration	
	χ_3_^2^	*P* value	χ_3_^2^	*P* value	χ_3_^2^	*P* value
Walking	43.1	<0.001	423	<0.001	11.5	*0.*009
Still	11.4	0.010	15.3	0.002	8.41	0.038
Flying	0.72	0.700*	97.3	<0.001*	0.48	0.815*
Upside down	14.6	0.002	64.5	<0.001	14.1	0.003
Grooming	1.96	0.374*	15.6	<0.001*	5.85	0.054*

* The 1 μM bees were excluded because they did not exhibit this behaviour

## Discussion

The data we present here shows that field realistic concentrations of 10 nM (i.e. 2–3 ppb) doses of neonicotinoids are sublethal to honeybees and are readily consumed by forager honeybees. Twenty-four hour exposure to a sublethal dose has subtle effects on motor function behaviour that are not readily seen by simple observations. The most common response to neonicotinoid exposure was more time spent grooming and an impairment of the righting reflex that lead to more time spent upside down. The overall effect on behaviour depended on the type of neonicotinoid that the bees were exposed to and the dose.

An important aspect of research on the impact of pesticides is testing concentrations that are relevant to the doses experienced by bees in the field. In our experiments, a 10 nM concentration is within the range of neonicotinoids reported from the nectar and pollen of seed treated plants (reviewed in Blacquiere et al. 2012; Rortais et al. 2005). Of the three most commonly used neonicotinoids, imidacloprid has been reported with the lowest concentration range (0.8–15 nM or 0.2–3.9 ppb), followed by clothianidin (7–10 nM or 1.8–2.5 ppb), and finally by thiamethoxam (22–34 nM or 6.6–10 ppb) (Schmuck et al. 2001; Blacquiere et al. 2012; Pohorecka et al. 2012). During 24 h of exposure, our methods mimicked the short-term exposure that foragers might get from floral nectar if they were foraging continually from a monoculture of plants that were seed treated with neonicotinoids for a 24 h period or eating contaminated honey within a hive. Furthermore, our data show that bees willingly feed on sugar solutions containing sublethal doses of neonicotinoids, perhaps indicating that they do not find these substances distasteful or cannot detect them. This indicates that they would feed on contaminated honey or nectar, even though concentrations as great as 100 nM could kill them. Further research is necessary to test this.

A recent statement has estimated that the nectar of seed-treated plants contains an ‘average maximum value’ of 1.9 ng/g of neonicotinoids (Godfray et al. 2014). The authors of this study predict that bees are exposed to a maximum dose of ~0.243 ng/day based on an estimated consumption of 128 mg/bee/24 h (~0.113 ml when adjusted for solution density). In contrast, we found that the bees in our cohorts consumed an average of 0.156 ml/24 h; this figure matches the amount we previously measured from individual forager honeybees in freely-moving cohorts fed 1 M sucrose solutions in our lab (Paoli et al. 2014). Importantly, this measured value reflects what foragers confined to a plastic box consume over 24 h and does not reflect the amount needed for flight. Foraging honeybees have a high demand for ATP which they derive from the catabolism carbohydrates and proline; their resting metabolic rate increases by +50-fold during flight (Joos et al. 1997). To obtain sufficient ATP, they would be required to eat more nectar. This would mean our estimates for mean daily consumption per bee are less than what flying foragers actually consume, implying that their exposure levels in the field could be as much as an order of magnitude greater than the measurements in Table 1. For these reasons, our data indicate that Godfray et al. (2014)'s calculation of the estimated exposure of foraging honeybees to neonicotinoids in seed treated plants is likely to be substantially lower than the actual amount bees obtain from floral nectar.

Our data show that 10 nM concentrations of neonicotinoids—doses ranging from 0.45 to 0.54 ng/bee—affect bee motor function mainly by disruption of the righting reflex and causing more grooming behaviour. In our assay, the control bees spent the most time walking. They rarely failed to right themselves when they fell over, and spent little time still or grooming. When bees were exposed to 10 nM doses of neonicotinoids in food, we did not observe a significant change in walking, flying and remaining still compared to the control. Only high concentrations of imidacloprid (e.g. 1 μM, 260 ppb), reduced walking behaviour; these bees did little besides sitting still. This is consistent with a previous report that showed that bees fed acutely or ad libitum for 24 h with very high doses (100–500 ppb) of imidacloprid spent more time stationary and less time walking or running (Medrzycki et al. 2003). Another study found that locomotion in bees depended on the dose administered: bees treated topically with an acute dose of 1.25 ng were hyperactive, whereas bees topically treated with >2 ng spent more time immobile (Lambin et al. 2001). Like the Lambin et al. (2001) study, we also observed that high doses of imidacloprid reduced walking behaviour. The imidacloprid dose in our study was 0.453 ng/bee—an amount approximately half that of the lowest dose used by Lambin et al. (2001). This difference could explain why we did not observe a ‘hyperactive’ phase. However, the means of administration were different in both studies, and this could have also influenced the outcome of both experiments.

When imidacloprid is consumed in sucrose solution, it takes ~6 h for bees to fully metabolise it (Suchail et al. 2004). Even within 20 min of ingestion, the overall concentration of imidacloprid found in bees is ~70 % of the fed dose, suggesting they metabolize it quickly. Using this estimate, we predict that the dose the nervous system of the bees in our study received when fed a 10 nM concentration was about 7 nM. Because the bees can metabolise neonicotinoids relatively rapidly, we expect that the ng/bee values we calculated in Table 2 are inflated, and that the actual value of each substance insulting the nervous system was lower. This same dose (10 nM) also impairs learning and memory in forager honeybees when bees have been exposed to it for 4 days (Williamson and Wright 2013). However, the effect of imidacloprid on learning and memory is not permanent; bees fed imidacloprid for 3 days and then given sucrose without imidacloprid for 3 days afterwards returned to normal functioning (Williamson and Wright 2013). For this reason, we predict that the motor function of honeybees would also return to normal within 24 h after imidacloprid was withdrawn from food.

The direct effects of the other neonicotinoids on bee motor function has not previously been investigated, although thiamethoxam and clothianidin have been implicated in impaired foraging and homing abilities. Clothianidin was reported to increase flight times during both foraging and homing at acute doses of ≥0.5 ng/bee, an effect also seen with imidacloprid at doses exceeding 1.5 ng/bee (Schneider et al. 2012). Thiamethoxam at a dose of 1 ng/bee has also been implicated in impaired homing ability, measured as a loss of foragers which failed to return to the hive (Henry et al. 2012). In light of the data we present here, it is interesting to speculate that impaired foraging and homing abilities in neonicotinoid treated bees may be partly due to a loss of co-ordination when performing motor function behaviours.

Overall, the effects of nicotine and the different neonicotinoids on motor function were similar in our experiments: the amount of locomotion performed was unaffected, but the ability to co-ordinate this locomotion and perform the righting reflex after falling over was impaired. A lack of co-ordination and an inability to recover correct posture after falling could have serious implications for the fitness of honeybees in a field setting: it is possible that reports of impaired foraging (Yang et al. 2008) and homing ability (Henry et al. 2012) in neonicotinoid treated bees could be partly due to timing and coordination of motor function, in addition to the more widely reported impaired learning and memory abilities (Decourtye et al. 2004a; Williamson and Wright 2013). The similar effects on co-ordination that we observed in bees treated with both nicotine and neonicotinoids is consistent with the idea that nicotine and neonicotinoids share a similar sites of action in the bee nervous system: the nicotinic acetylcholine receptors (Brown et al. 2006; Buckingham et al. 1997). However, different sub-populations of nicotinic receptors have different pharmacological profiles: although all by definition are activated by nicotine, only a sub-population is sensitive to imidacloprid (Gauthier et al. 2006).

Interestingly, thiamethoxam and nicotine exposure also increased grooming behaviour. Increases in grooming behaviour have also been reported in honeybees treated with other neurotoxic substances, using the same motor function assay. For example, both ethanol, and the organophosphate acaricide, coumaphos, caused bees to engage in more grooming behaviour (Maze et al. 2006; Williamson et al. 2013). We expect that the motor circuits governing grooming behaviour are mediated by specific receptors mediating cholinergic neurotransmission in the ventral nerve chord. Ligand-binding studies using radiolabelled neonicotinoids indicate that this may indeed be the case: imidacloprid binds to the same site as α-bungarotoxin, but thiamethoxam has a lower affinity for the α-bungarotoxin sensitive receptor subtype (Wiesner and Kayser 2000). Instead, it appears that thiamethoxam has additional binding sites within the nervous system not shared by imidacloprid (Wellmann et al. 2004). The presence of different receptor subtypes in the nervous system can also explain why the observed effects of neonicotinoids on locomotion were overall quite subtle, compared to the more dramatic effects of neonicotinoids on olfactory learning and memory (Decourtye et al. 2004b; Williamson and Wright 2013) and brain function (Palmer et al. 2013). It is known that imidacloprid-sensitive nicotinic receptors are abundant in the brain areas involved in olfactory learning and memory (Barbara et al. 2008; Deglise et al. 2002), but the distribution of neonicotinoid sensitive receptors in the ganglia of the ventral nerve cord and the neuromuscular junctions has not yet been studied in the honeybee.

In conclusion, the data we present here adds to the body of work suggesting that field-realistic concentrations of neonicotinoids have subtle behavioural effects on honeybees, which could impair ecologically relevant behaviours such as foraging during a short term exposure of 24 h, and ultimately reduce colony fitness. Based on unpublished data from our laboratory, we expect that our results for bees exposed to neonicotinoids for 24 h reflects how they behave when exposed for several days. The difference in our studies with field-relevant exposure of forager honeybees is that our bees were not in flight. It is possible that flight would require that bees consume more solution, and hence receive a bigger dose of the pesticide—perhaps resulting in stronger effects on motor function. Such subtle behavioural effects should be taken into account when pesticides are tested for ecotoxicity. Tests, like the behavioural observations we report here, would be a rapid means of assessing the impact of longer-term exposure to pesticides on bee motor function and could be used as a reliable bioassay for sublethal effects on pollinators.
